# Electrophysiological, emotional and behavioural responses of female targets of sexual objectification

**DOI:** 10.1038/s41598-023-32379-w

**Published:** 2023-04-08

**Authors:** Bianca Monachesi, Alice Deruti, Alessandro Grecucci, Jeroen Vaes

**Affiliations:** 1 Department of Psychology and Cognitive Science, University of Trento, Trento, Italy; 2 Centre for Medical Sciences, CISMed, University of Trento, Trento, Italy

**Keywords:** Social neuroscience, Emotion, Social behaviour

## Abstract

Sexual objectification and the interiorized objectifying gaze (self-objectification) are dangerous phenomena for women’s psychological wellness. However, their specific effects on women’s socio-affective reactions are still poorly understood, and their neural activity has never been explored before. In the present study, we investigated women’s emotional and electrophysiological responses during simulated computer-based objectifying social interactions, and we examined consequent punishing behaviours towards the perpetrator using the ultimatum game. Behavioural results (N = 36) showed that during objectifying encounters women generally felt angrier/disgusted and tended to punish the perpetrator in later interactions. However, the more the women self-objectified, the more they felt ashamed (*p* = 0.011) and tended to punish the perpetrators less (*p* = 0.008). At a neural level (N = 32), objectifying interactions modulated female participants’ neural signal elicited during the processing of the perpetrator, increasing early (N170) and later (EPN, LPP) ERP components. In addition, only the amplitude of the LPP positively correlated with shame (*p* = 0.006) and the level of self-objectification (*p* = 0.018). This finding provides first evidence for the specific time-course of sexual objectification, self-objectification and its associated shame response, and proves that emotional and social consequences of sexual objectification in women may depend on their tendency to self-objectify.

## Introduction

Interpersonal encounters and social interactions represent one of the most common ways through which women are treated and perceived focusing on their physical appearance and on the aesthetic of their body parts, that is, as sexual objects^1^. Especially in Western societies, instances of sexual objectification such as men’s sexual cat calls, wolf whistles, sexual remarks, and sexist stereotypes occur to a woman about once every other day^2^. According to Objectification theory^1^, women who repeatedly become the target of sexual objectifying events may manifest a further phenomenon known as self-objectification^3,4^. Whenever women self-objectify, they internalize the observer’s objectifying perspective, meaning that they start judging themselves with a focus on their physicality. Both sexual- and self-objectification have adverse consequences on emotional and social dimensions in a woman’s life^1,3^. These consequences are harsher for women rather than men^4,5^, and have been related with various psychopathological outcomes (e.g., depression and eating disorders,^6,7^). Therefore, getting a better understanding of these toxic interpersonal dynamics is of utmost importance. In the present study, we sought to investigate the direct effects of sexual and self-objectification on women’s emotional responses, and later behaviours towards the perpetrator. Most importantly, for the first time we also explored modulations of women’s neural activity during these objectifying encounters with men.

In the literature, there is growing evidence on the adverse emotional and interpersonal consequences in female targets of sexual objectification. Nevertheless, the way they may be related to each other is still unclear. Because of higher body surveillance or monitoring of the body’s appearance^1^, self-objectification has been usually associated with increased stress, anxiety, guilt, and shame toward one's own body^4,6–10^. Especially shame is a self-conscious emotion that involves self-blaming, and that seems to play a pivotal role in consequent social interactions. Such interactions have been traditionally associated with withdrawal behaviours and interpersonal problems^11–13^. Some studies found that when women self-objectify or become victims of sexual objectification, they report less engagement in social activism^14^, less relationship competencies^4^, less optimal intrinsic motivational states (i.e., flow,^6^), and they perceive the objectifying man as less likeable^15,16^, showing less willingness to affiliate with him or reducing their verbal interaction time^5,15,16^, (but see^17^).

Alongside this literature, however, there is also evidence that female victims of sexual objectification report hostile intent and aggressive/punishing behaviours toward the objectifying men^18^. After the exposure to verbal stranger harassment, for example, some authors found that body shame through body surveillance increased, but only in women who reacted to the harassment with happiness, empowerment, or—interestingly, with low levels of anger^19^. In a complementary way, it has been reported that women in interaction with an objectifying man felt more dehumanized and experienced more anger and sadness^20^. Victims of dehumanization report more negative emotions (anger, sadness, shame and guilt,^21^) and higher levels of aggressive tendencies^22^. Differently from shame, negative emotions such as anger or disgust have been usually associated with more active and aggressive behaviours (e.g.,^23^), in line with the hypothesis of a tight relationship between emotions and action tendencies^24^.

Attempting to bring order to these apparent mixed findings, it seems reasonable to expect that, in sexual objectifying contexts: women experiencing shame tend to act more passively and interiorize an objectifying gaze towards the self, whereas women experiencing anger/disgust tend to react more aggressively. In support of this idea, two recent studies^25,26^ reported that among female victims of sexual objectification, anger and disgust positively predicted potential active responses toward the perpetrator, whereas shame predicted self-blame responses. Although these findings provide initial evidence on the relationship between emotional responses and interpersonal consequences, the authors overlooked an aspect we hypothesize can be implicated in women’s shame or anger responses. This aspect concerns the kind of objectifying experience and the victim’s reaction that is considered. Indeed, in many studies in which shame was reported, the authors correlated this response with the measure of self-objectification, assessed by self-reports (e.g.,^4,6–9^). Instead, when authors manipulated sexual objectification in a between-subjects experimental design, women who were sexually objectified (but who did not self-objectify) reported more anger responses (e.g.,^18,20,25^). These considerations lead us to argue that the specific emotional and social consequences of sexual objectification may depend on the tendency to self-objectify.

No previous research directly explored whether the type of emotion experienced and the consequent behaviour enacted by women may be traced to the increased tendency of women to self-objectify during sexual objectifying interpersonal encounters. The present study aims to bridge this gap, not only manipulating sexual objectification and including a self-objectification index, but also extending Shepherd’s^25^ findings by the investigation of both emotional and behavioural responses in a more direct and ecological way (see below). Even more importantly, the present study analyses women’s reactions to an objectifying interpersonal dynamic through a neural perspective. Indeed, whether and how sexual objectifying contexts modulate women’s neural responses during interactions with men who objectify them has never been investigated. Such a perspective, however, is promising, especially because the way socio-affective contexts modulate the time course of others’ neural processing has gained increased interest in the last decades, especially when associated with the typical face-in-context paradigm^27^. The scientific advancements using this approach generally relied on three main methodological aspects: verbal statements to manipulate the context; individuals’ faces to symbolize the “other”, and the electroencephalography technique that with its high temporal resolution allowed researchers to investigate the serial affective and cognitive processes indexed by the typical face- and emotion-based Event-Related-Potentials (ERPs).

Several studies showed that positive or negative person’s knowledge/behaviours conveyed by verbal descriptions represent a potent means to change the salience of an associated neutral face, and result in modulations of early and later ERP components^28–36^. The typical early ERP is the N170, a negative wave that reaches its maximum amplitude between 130 and 220 ms in the parieto-occipital sites, usually of the right hemisphere (e.g.,^37,38^). The N170 has been traditionally linked to the initial processing of the structural/configurational information of faces^39^. Although previous studies reported that affective (emotional expressions vs neutral expressions) and self-relevant (frontal vs averted gaze) perceptive features of faces increase the N170 amplitude^38,40,41^, the effect of socio-affective contexts on this early component is still mixed. Indeed, some studies reported that the emotional context increases the amplitude of the N170^28,31,40^, whereas other studies failed to find a similar modulation^29,34,35^. Besides the N170, other relatively early and later ERP components have been proposed as more direct indicators of facilitated emotion processing during the presentation of faces in a context. Specifically, the early posterior negativity (EPN) is a negative wave occurring in parieto-occipital sites, between 200 and 350 ms^30,32^ and is involved in the increased attention towards emotional stimuli^42^. The late positive potential (LPP), instead, is a centro-parietal positive wave which can start at 300/500 ms after stimulus onset and can be sustained up to 800 ms^36^ or more^41,43^. The LPP reflects more elaborate cognitive evaluation of the emotional content and its meaning in a motivated attention framework^44^. For what concerns these ERPs, there is much more consistency among studies about the direction of their modulation by socio-affective contexts during the processing of neutral faces. Increased amplitude of both EPN and LPP is reported for faces associated with verbal descriptions about a person’s biography/behaviours, especially when negative (e.g.,^28,30,32–35^, but see^31^ for the effect of positive contexts on LPP). Therefore, the “face-in-context paradigm”^27^, and the ERPs analysis seem to perfectly fit the intent to investigate neural responses of women facing others associated with the specific socio-affective context of sexual objectification. Indeed, the advantages of this approach are two-fold: first, we will be able to describe the affective and cognitive processes involved in the perception and in the interpretation of encounters with men who sexually objectify female targets, especially in relation to women’s tendency to self-objectify in such situations. Second, the real-time recording of participants’ neural activity will help us to overcome the well-known drawback of self-report measures that do not always reflect participants’ intimate thoughts and feelings due to a lack of introspection or social desirable responses.

To sum up, the present study aimed at better understanding the phenomenon of female sexual objectification. Namely, we focused on how interactions with men who sexually objectify female participants or not trigger specific emotional responses, modulate the time course of neural responses, and increase punishing behaviours towards the perpetrator. In addition, the influence of the attention towards one’s own body/appearance (as indexed by self-objectification) during objectifying interactions on all these measures will be investigated as well. To these aims, participants performed three consecutive tasks. The first one, the Objectification Task (OT), was a typical face-in-context paradigm adjusted to build a more ecological objectifying encounter with men. The socio-affective context in the OT preceded the presentation of a man’s face, and consisted in an objectifying or non-objectifying sentence. Participants were told the man’s face represented the person pronouncing the sentence. After each presumed interpersonal encounter, female participants reported the emotion they experienced (among neutral, anger, shame and disgust emotion), and the extent to which they shifted their attention towards their own physical appearance (on a 4-point Likert scale). As such, during the OT, the objectifying context was manipulated and the measure of self-objectification was assessed after each encounter. In this first phase, the EEG signal was recorded in order to investigate the effect of the context on the processing of the objectifying other. The successive task, the Who Says What (WSW), was used to check the level of association between the man’s ID and the objectification condition during the OT task (because of space constraints, details of the procedure, data analysis and results of this task are reported in the Supplementary online material S1). Finally, participants performed the Ultimatum Game task (UG,^45^) to assess punishing behaviours toward the previously encountered men. Using a common version of this game (e.g.,^46^), the subject decided whether to accept or reject a money partition (received from the same men they “met” in the previous tasks), that can be fair or unfair (i.e. when the bidder wins more money). Since the rejection scenario implies a loss of money for both parties, the performance at the UG has sometimes been interpreted as an index of punishment towards the bidder^47^. Precisely for this reason, this paradigm was appropriate to investigate whether punitive behaviour changes depending on whether the offer came from an objectifying man or not. No EEG signal was recorded during the latter task.

Behaviourally, we expected that the objectifying interpersonal context, relative to the non-objectifying one, would increase participants’ focus on their physical appearance, as well as their negative emotional responses in general, and anger or disgust in particular. In addition, the objectifying interpersonal encounters with men will increase the punishing behaviour towards them. In neural terms, the objectifying context, due to its link with specific negative responses, is expected to increase the amplitude of early (N170) and later components (EPN and LPP). Besides these results, we also expect that self-objectification (indexed by the level of focus on physical appearance) will influence the above measures in the way that higher self-objectification will induce more shame responses and less punishing behaviours. Because this is the first time that neural measures are investigated during objectifying encounters, we have no specific predictions concerning the effect of self-objectification on the EEG signal. However, we can expect a modulation on later ERPs in line with previous evidence showing their relation with self-relevant emotional information^29,34^.

## Results

### Objectification task

#### Behavioural results

The t-test between the mean levels of self-objectification was significant, *t*(35) = 13.09, *p* < 0.001, *d* = 2.18, showing that in the objectifying condition (*M* = 2.52; *SD* = 0.55) self-objectification was greater than in the non-objectifying condition (*M* = 1.75; *SD* = 0.34).

**Figure 1:**
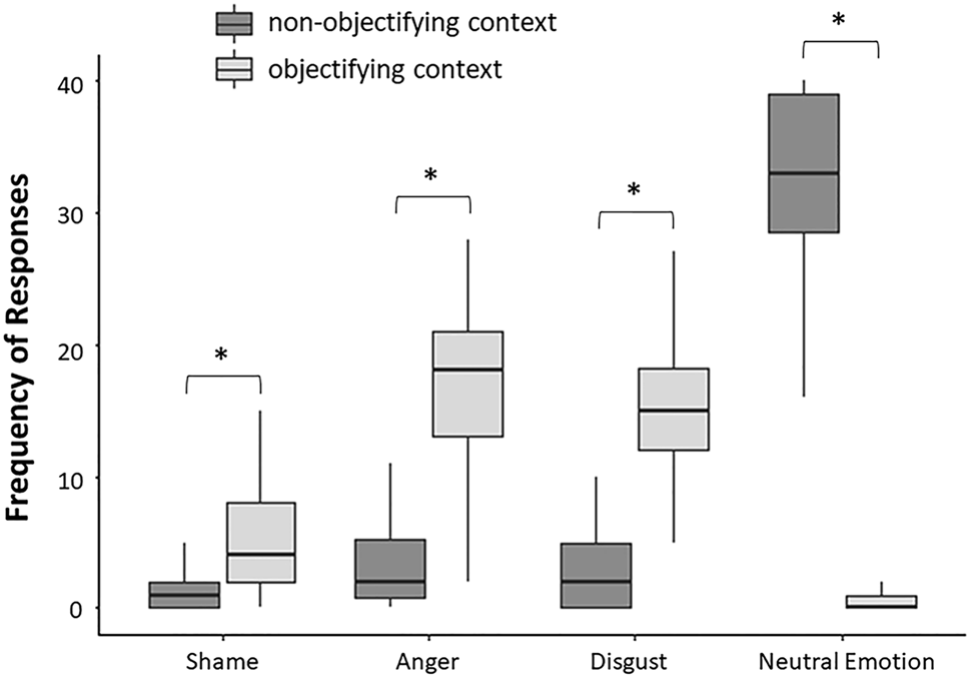
Behavioural results of the objectification task. Frequency of responses as a function of emotion (shame, anger, disgust and neutral emotion) and objectification condition (objectifying and non-objectifying context). (*) indicates significant differences (*p* < 0.05).

ANOVA results showed a significant main effect of emotion, *F*(3,105) = 60.74, *p* < 0.001, *η*_*p*_^*2*^ = 0.63, revealing that the frequency of neutral emotion (*M* = 16.54, *SD* = 4.27) was the highest, *ps* < 0.001, followed by those of anger (*M* = 10.22, *SD* = 3.43) and disgust emotions (*M* = 9.54, *SD* = 3.23) that did not differ, *p* > 0.99, and with shame (*M* = 3.56, *SD* = 3.13) being the least frequent emotion, *ps* < 0.001. The main effect of emotion was qualified by a significant interaction with objectification (*F*(3,105) = 236.90, *p* < 0.001, *η*_*p*_^*2*^ = 0.87). As expected, pairwise comparisons showed that the mean frequency of the three negative emotions in the objectifying condition were significantly greater than those in the non-objectifying context (Shame: *M*_*obj*_ = 5.33, *SD* = 4.89, *M*_*Non-obj*_ = 1.78, *SD* = 2.64; Anger: *M*_*obj*_ = 17.4, *SD* = 5.96, *M*_*Non-obj*_ = 3.00, *SD* = 3.17; Disgust: *M*_*obj*_ = 15.86, *SD* = 5.99, *M*_*Non-obj*_ = 3.22, *SD* = 4.18), all *ps* < 0.001. While the average of the frequency of neutral emotions was significantly greater in the non-objectifying (*M* = 31.86, *SD* = 1.33) compared to the objectifying condition (*M* = 1.22, *SD* = 0.43), *p* < 0.001 (see Fig. 1).

#### Neural results

##### N170

**Figure 2:**
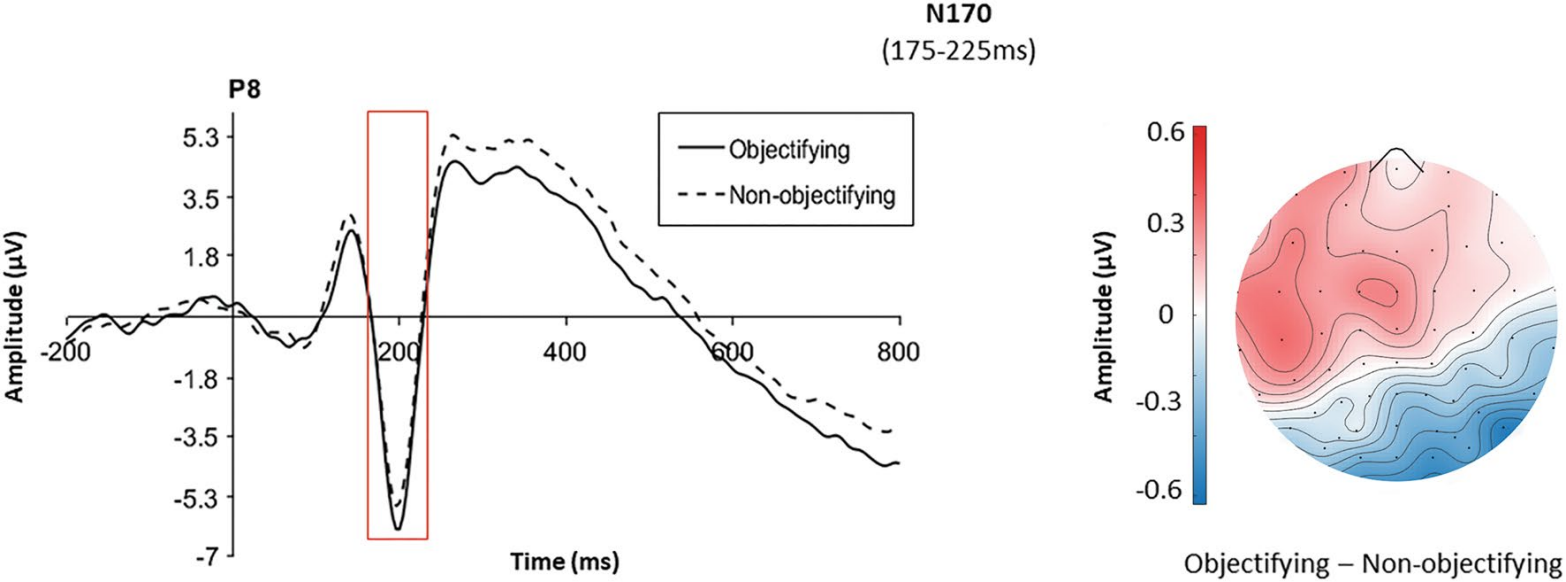
Waveform and scalp distribution of the N170. Waveform (left of the panel) is plotted as a function of the objectification at the right PO8. Scalp distribution (right of the panel) displays the difference between objectifying and non-objectifying condition in the time window 175–225 ms.

The ANOVA performed on N170 showed a significant main effect of the lateralization factor, *F*(1,31) = 6.17, *p* = 0.019, *η*_*p*_^*2*^ = 0.17, with the component amplitude being higher in the right hemisphere (*M* = − 4.00, *SD* = 1.04) than in the left one (*M* = − 1.99, *SD* = 0.76). The main effect of objectification was also significant, *F*(1,31) = 4.95, *p* = 0.034, *η*_*p*_^*2*^ = 0.14, with the N170 amplitude being higher in the objectifying (*M* = − 3.17, *SD* = 0.81) than in the non-objectifying condition (*M* = − 2.82, *SD* = 0.82). The lateralization*objectification interaction was significant, *F*(1,31) = 4.84, *p* = 0.035, *η*_*p*_^*2*^ = 0.14, demonstrating that the main effect of objectification was significant in the right, *p* = 0.008 (*M*_*obj*_ = − 4.28, *SD* = 5.85, *M*_*Non-obj*_ = -3.72, *SD* = 5.91), but not in the left hemisphere, *p* = 0.406 (*M*_*obj*_ = − 2.06, *SD* = 4.32, *M*_*Non-obj*_ = − 1.92, *SD* = 4.37) (see Fig. 2).

##### EPN

**Figure 3:**
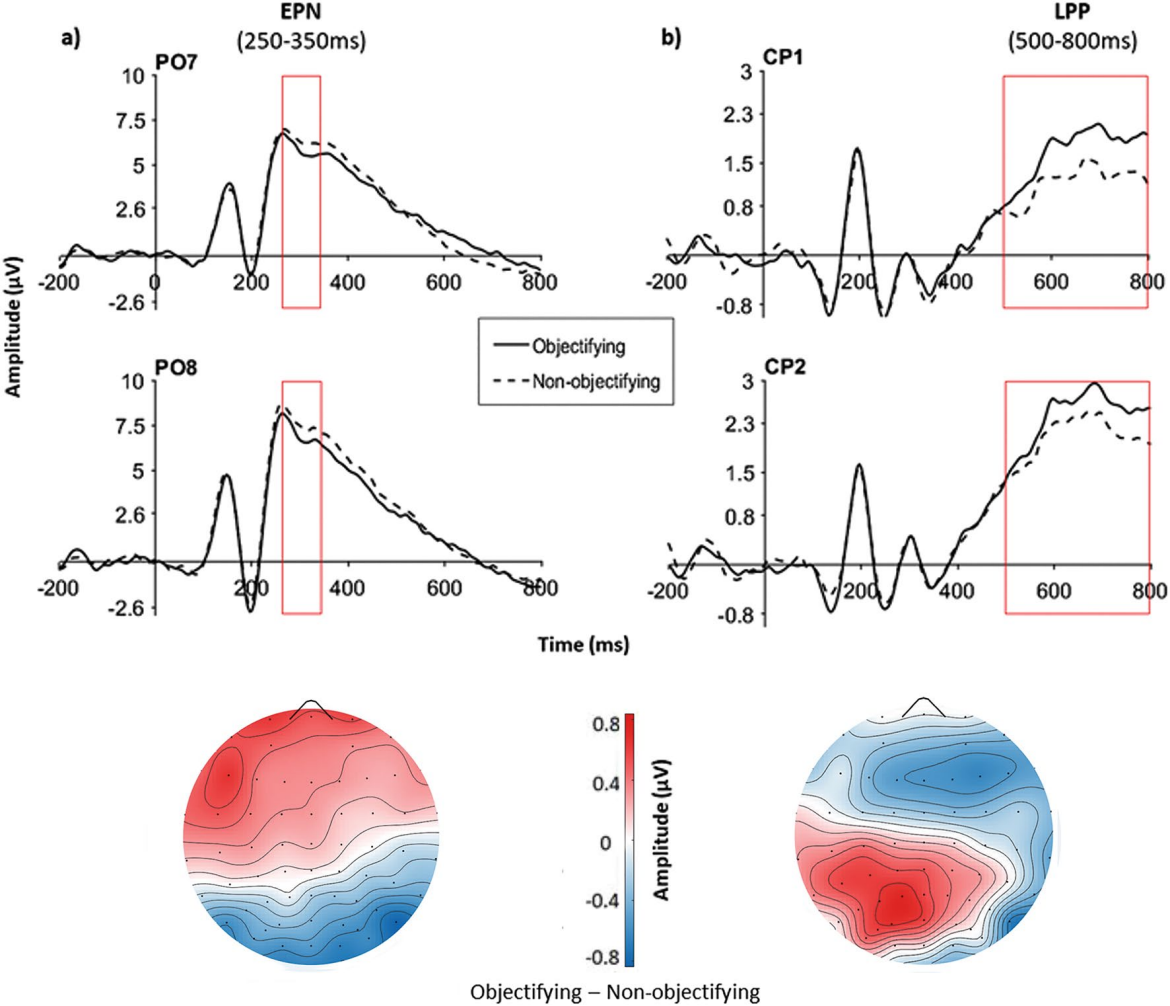
Waveforms and scalp distributions of the EPN and LPP. (**a**) EPN, and (**b**) LPP components. Waveforms (up of the panel) for the two components are plotted as a function of the objectifying and non-objectifying contexts in two representative electrodes: PO7-PO8 and CP1-CP2 for EPN and LPP, respectively. Scalp distribution (bottom of the panel) for the two components represents the difference between the objectifying and the non-objectifying conditions in the relevant time window of each ERP.

The t-test comparing the EPN component in the objectifying and in the non-objectifying condition was significant, *t*(31) = 3.71, *p* = 0.001, *d* = 0.66 showing that the EPN amplitude in the non-objectifying condition (*M* = 5.70, *SD* = 5.06) was smaller than in the objectifying contexts (*M* = 5.10, *SD* = 4.90) (see Fig. 3a).

##### LPP

Also the t-test comparing the LPP component in the objectifying and the non-objectifying conditions was significant, *t*(31) = 3.00, *p* = 0.005, *d* = 0.53 revealing that the LPP amplitude in the objectifying condition (*M* = 2.08, *SD* = 1.57) was higher than in non-objectifying contexts (*M* = 1.63, *SD* = 1.61) (see Fig. 3b).

### Ultimatum game task

The t-test comparing the frequencies of rejection in the objectifying and in the non-objectifying condition was significant, *t*(35) = 3.29, *p* = 0.002, *d* = 0.54 revealing that the frequency of rejection in the objectifying condition (*M* = 6.78, *SD* = 1.87) was higher than in the non-objectifying context (*M* = 5.92, *SD* = 1.71).

Further analysis reported in the Supplementary online material (S2) confirmed that the effect occurred especially for those men that had been correctly associated to the objectification condition in the WSW. This suggests, then, that punishment behaviours were explicitly and consciously addressed to objectifying men.

### Correlation analyses

**Figure 4:**
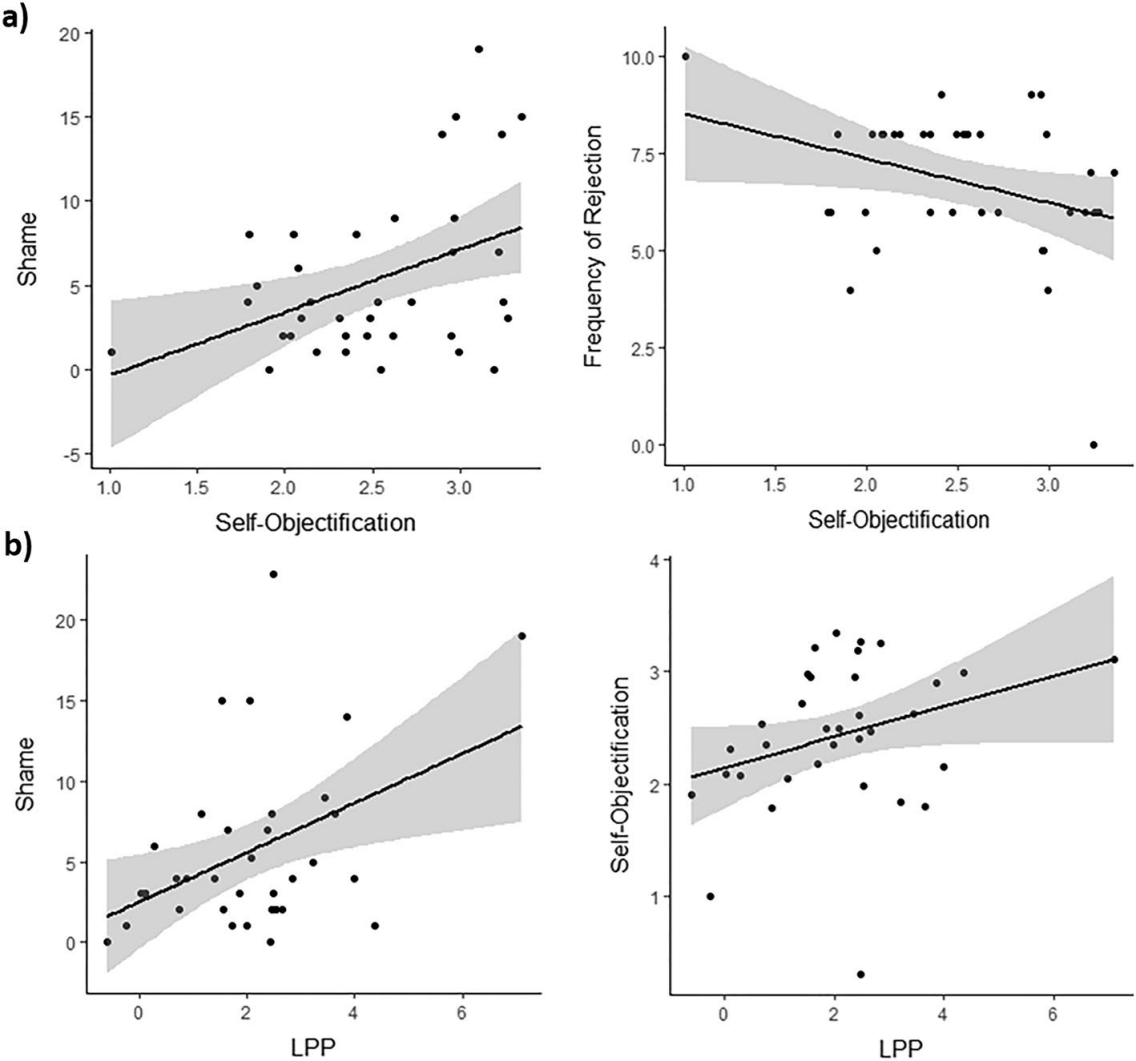
Results of correlational analyses. (**a**) Correlation between the level of self-objectification and the shame emotion, on the left, and the frequency of rejection, on the right; (**b**) correlation between the amplitude of the LPP and the shame emotion, on the left, and the level of self-objectification, on the right.

The first analysis showed a significant positive correlation, *r*(35) = 0.42, *p* = 0.011, between the level of self-objectification and the frequency of shame responses (see Fig. 4a, on the left). Furthermore, a significant negative correlation, *r*(35) = − 0.33; *p*_uncorr_ = 0.046, emerged between the level of self-objectification and the frequencies of rejection (see Fig. 4a, on the right). Although this latter significance did not survived FDR correction, we performed the correlation also with the frequency of rejection when referred to the objectifying men correctly remembered by participants in the WSW (see S4), and the effect was stronger as well as correction-resistant, *r*(35) = − 0.43; *p* = 0.008.

Results of the second correlation showed that LPP amplitude positively correlates with both the self-objectification level (*r*(31) = 0.42; *p* = 0.018) and the frequency of Shame responses (*r*(31) = 0.48; *p* = 0.006) (see Fig. 4b). Instead, no correlations were found for the N170 and the EPN component (*ps* > 0.11).

To assure that the significant correlations and the related effects were specific to the objectification manipulation, we performed additional correlational analyses among the same variables, all in the non-objectifying condition. We failed to find the same patterns (results of these analyses are reported in the Supplementary online material S3), supporting that the effects are specifically due to the objectification manipulation.

## Discussion

The widespread phenomenon of sexual objectification may represent a potential menace for women, in terms of emotional and social functionality. Only in the last few years, its investigation encompassed the use of neuroscientific methodologies to enrich the knowledge about the still unknown underlying mechanisms and consequences of this phenomenon. Although this new approach has mostly focused on the comprehension of the neural correlates underpinning the objectifying gaze (e.g.,^48,49,70^), no interest has been addressed to the study of the neural correlates related to the objectified woman’s perspective. With the aim to bridge this gap, in the present study we used a paradigm which allowed us to simulate computer-based objectifying encounters between female participants and men, thought to be real persons (the OT). This interpersonal encounter consisted in the presentation of an objectifying or non-objectifying sentence, associated with a male’s face who allegedly pronounced this phrase toward the participant. During this task, for each social interaction presented, we recorded the women’s emotional response, the level of self-objectification and, importantly, the electrophysiological signal related to the processing of the male targets’ faces. In a following task (WSW), the same faces were presented with both an objectifying and a non-objectifying sentence, in order to check whether participants learnt the association between each man and the type of sentences he pronounced. Finally, we investigated the punishing behaviour of women towards the objectifying compared to the non-objectifying men in an ultimatum game.

Behavioural results showed that the objectifying context manipulation in the OT occurred successfully, and the expected attentional shift on one’s own physical appearance increased when participants interacted with a man who objectified them. When participants were victims of sexual objectification, they also reported to experience more negative emotions, especially anger and disgust, followed by shame. In the following UG task, participants more often rejected money offers especially received by those men who participants explicitly recognized as objectifying.

Results in the neural analyses showed that the objectifying context modulated neural responses from the early to the later ERPs. Indeed, the mean amplitude in the N170, EPN and LPP components increased when women interacted with objectifying, relative to non-objectifying men. Interestingly, correlational analyses helped us to better understand how sexual objectification impacts interpersonal encounters in terms of women’s emotional, behavioural and neural responses. Although anger and disgust were the most reported emotions, the level of self-objectification correlated only with the expression of shame, such that higher levels of self-objectification were linked with more shame responses. In addition, both the level of self-objectification and shame responses increased as the mean amplitude of the later LPP components were larger. Finally, the level of self-objectification also negatively correlated with the frequency of rejections during the UG, suggesting that participants who reported to self-objectify more, were less inclined to punish those who objectified them. These results have relevant implications for both of the central topics of the current study: the phenomenon of sexual-objectification, and the neural mechanisms underlying perceptions of individuals embedded in socio-emotional contexts.

For what concerns sexual objectification, our findings clearly suggest that female targets of sexual objectification react following two distinctive emotional and behavioural paths. Indeed, our findings concur with the apparently divergent literature that sometimes reported increased shame^4,6–9^ and sometimes increased anger/disgust in sexually objectifying contexts^20,25,26^; at the same time these contexts sometimes elicited active^18,25,26^ and sometimes passive actions^5,14–16^ towards the objectifying men. The present findings suggest that female targets of sexual objectification generally experience anger/disgust and actively respond to the perpetrator, punishing him. Still, those women who self-objectify more, also experience more shame emotions and punish the perpetrator less. The increased punishment behaviour in female targets of sexual objectification is consistent with previous studies in which objectified women reported higher willingness to activism^26^, or increased explicit aggressive behaviours^18^. More generally, anger responses are typically related to aggressive behaviours^23^ or direct aggression^50^, making it reasonable that women act in this way when they experience anger during an objectifying social interaction. In addition, based on the fact that the emotion experienced usually reflects the individual’s interpretation of the situation^51^, anger is likely when a moral violation is appraised^52^, especially when this violation menaces the self^50^, much like what typically occurs in sexually objectifying contexts.

Less punishing behaviour or the tendency to accept the objectifying men’s offers more (even when unfair) is in line with previous studies as well, although it can be interpreted in two different ways. When women self-objectify, indeed, they can adopt an identity as if they were an object, speaking less^5,15^, and being more passive^14^. Slightly different, self-objectified women can attempt to be accepted by others by being more compliant^17,19^. In support of both interpretations, shame is linked with worries over one’s own self-image and it may yield the willingness to hide as well as to repair one’s own image^53^. Although the reasons why women who self-objectify reacted in this way go beyond the scope of the current experimental effort, future studies can help to extend our findings in this sense.

Interestingly, the results relative to the neural time course of face processing during objectifying encounters support and enrich the above findings in several ways. They clarify the large impact sexual objectification has on women. Objectifying contexts showed to modulate already the early step (N170) of a person’s processing, when only structural or configural aspects of the face are supposed to have an impact^54^. All men’s faces in this study were neutral at the beginning, both in terms of inner perceptive features (i.e., no emotional expression), and of the associated socio-affective information. It means that objectifying encounters trigger a neural response in women resembling that of explicitly negative faces^38^. In the face-in-context literature^27^, these results are partially in line with previous studies, even though some of them^29,35^ failed to report modulations of the N170 as a function of the affective context. The results in the latter studies are probably due to methodological aspects which reduced or confused the effect. Some examples include the fact that the EEG signal was recorded in a successive face recognition task and not during the face-context association task^35^, or the use of coloured, instead of B/W photos^29^, a perceptive detail that largely affects the N170 (e.g.,^55^). Importantly and in line with our findings, some other studies found increased N170 for neutral faces preceded by negative personal information about the individuals^28,30,31^. In addition, it is worth noting that the N170 was also found to be sensitive to other social information such as own-age, own-race, gender stereotypes and social categorization (e.g.^56–58^). Therefore, the present findings not only suggest a rapid effect of the objectifying context on the time-course of face processing, but also provide further evidence that the modulations of this early ERP may go beyond perceptive facial features.

The neural results were also crucial in supporting differences between sexual and self-objectification in terms of their affective and neural consequences. Sexually objectifying contexts generally increased ERP amplitudes from the N170 to the LPP. Self-objectification, instead, was specifically associated with the LPP as well as the frequency of shame responses. Although our results do not allow us to directly disentangle whether the modulation of the neural activity related to sexual objectification was due to the impact of negative emotions and/or the social context, the information we can extract regarding the self-objectification phenomenon is particularly clear. The LPP represents a relevant ERP related to the top-down evaluation of emotional stimuli^44^ and many previous studies reported that negative contexts increased its amplitude during neutral face processing (e.g.,^35,37,40^). Importantly, the amplitude of this ERP increases also for self-relevant, relative to self-irrelevant emotional contextual information^29,34^, paving the way to create a link between the LPP and the two social and affective responses. Both shame and self-objectification, indeed, are strictly related to the evaluation of the self from a moral and a physical perspective, respectively. In addition, although there is evidence that shame can be rapidly distinguished from guilt at an early ERP (P200,^53^), it also elicited increased positive amplitudes, especially at later ERPs, relative for example to happy emotions^53^, a neutral control condition^59^, and in patients with social anxiety, in which shame is known to be high^60^. Unfortunately, the neural effect in these previous studies was not directly correlated to shame reports, making it impossible to track the time course of this emotion. Our study, instead, clarifies how the emotion of shame can be neurally traced and linked to the LPP, laying also a preliminary foundation on why it can be strictly related to self-objectification. Even if our finding can not be discussed in terms of causality, it is in line with and support the antecedents and consequences of self-objectification proposed by Fredrickson et al.^69^. Indeed, the authors suggest that sexually objectifying events (culture-related), like our verbal stimuli, may induce women to increase the appearance monitoring, or self-objectification, which in turn increases the chance to feel shame.

To better address further investigations in this research field, some limitations of the current study need to be pointed out. The emotions during the OT were recorded on the basis of their frequency but not in terms of their intensity on a Likert scale. This aspect could have provided further information about the strength of the felt emotions, which may have differed for negative as well as neutral responses. Related to this limitation, we allowed participants to select only one emotion among those proposed, which were all negative (anger, disgust and shame), except for the neutral one that was intended to be relevant for the non-objectifying scenarios. This choice was adopted according to the pre-validation phase of the verbal stimuli (see Supplementary online materials S4), during which positive emotions have almost never been selected. However, this choice prevented participants to freely select among a wider range of emotions, including some positive ones (see ^25^, for the involvement of positive emotion in sexual objectifying contexts). Moreover, because of the highly complex experimental session, we did not include in the experimental design a negative, but non-objectifying context. Adding such a condition might have helped to highlight any kind of overlap with other negative, social scenarios (e.g., racism). The distinction or overlap with other social and negative contexts was not the aim of the present study, but in light of its promising results, further investigations may control for this aspect to separate the impact of objectifying interactions from mere negative social interactions. Finally, the potential differences in self-relevance between the objectifying and the non-objectifying sentences were not directly controlled. Although the emotionality and the particular form of self-attention (i.e., self-objectification) seemed to have triggered the neural differences between the objectifying and non-objectifying conditions at later ERPs, the specific effects of emotionality, self-relevance, and self-objectification need to be investigated in more detail in the future.

To conclude, the present study represents an important step towards a better understanding of the phenomenon of sexual objectification, in terms of its affective, social and—most importantly, its neural consequences in targeted women. Especially the neural findings helped to shed new light on the mechanisms underlying the processing of neutral faces when embedded in socio-affective contexts. Our study provides evidence of a different neural-time course between sexual and self-objectification, with the latter showing a clear link with shame. Our findings support the idea that the tendency to self-objectify plays a crucial role in the way women respond to these specific social interactions. We hope these finding will be an input to the study of sexual objectification using a more ecological methodology and including more often neuroscientific techniques. Finally, we hope this study will be helpful for improving women’s awareness about this phenomenon and the different ways to face it.

## Method

### Participants

Thirty-six female participants took part in the study (age: *M* = 21.83, *SD* = 3.36). For the neural analysis, this sample size was consistent with previous studies using the ERP analysis and the face-in-context paradigm^28,29,31,32^. Similarly, a sample of minimum 31 participants was calculated a priori using G*Power (^61^, power = 0.85, α = 0.05) to obtain a moderate effect size of f = 0.25, for the emotion by objectification behavioural analysis. All participants were Italian native-speakers and reported normal or corrected-to-normal vision. All participants gave their written informed consent to take part in the study and all of them received a monetary compensation of 15€ for their participation. NB: Participants were told monetary compensation was 10€, plus up to 5€ according to their performance in the last task (the UG). The entire experimental procedure was carried out in accordance with the Declaration of Helsinki and approved by the ethical review board of the University of Trento.

### Stimuli and apparatus

The visual stimuli were made up of phrases and photos of male faces. Verbal stimuli consisted in 84 sentences, 42 objectifying and 42 non-objectifying. Objectifying sentences refer to “pick-up lines” or typical “sexist stereotypes” and, in both cases, focus was on woman’s aesthetic qualities. Non-objectifying sentences matched objectifying ones in syntactic and grammatical terms, and—importantly, they had no reference to aesthetic or physical aspects and tended to be as neutral as possible. All sentences were selected from an original set pre-validated (see Supplementary online materials S4 for the pre-validation procedure, the details of the selected stimuli, and examples of them).

Face stimuli were 20 B/W images of White males with neutral emotional expression selected from the Chicago Face Database (CFD,^62^). The faces were divided into two subsets, and balanced (on the basis of the CFD norming data) for luminance and several socio-emotional evaluations: attractiveness, reliability, masculinity, dominance, perceived emotions of anger and joy (all *ps* > 0.2 obtained with independent t-tests contrasting the two subsets).

Stimuli were presented on a DELL Precision 7710 Laptop with a 17″ screen Full HD. Tasks were developed using OpenSesame software^63^, which also recorded participants' responses, entered using a standard USB-keyboard.

### Task and procedure

**Figure 5:**
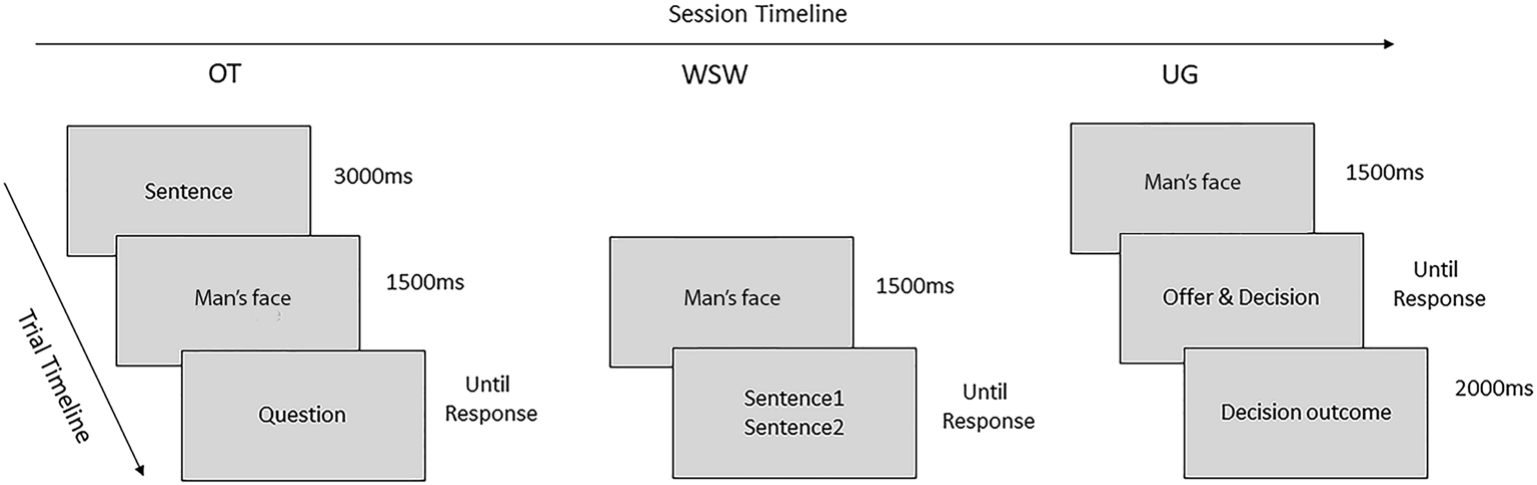
Experimental session and trial timelines. Order of the three tasks performed by the participants, and timeline of the trials for each of them. OT, objectification task; WSW, who says what; UG, ultimatum game task. For the WSW details see Supplementary online materials S1.

For details about the cover story and tasks instructions provided to participants see the Supplementary online material (S5). The OT was composed of 4 practice trials, and a total of 160 experimental trials divided in two blocks of 80 trials each. In each trial, a verbal stimulus was followed by the presentation of the male’s face. After that, participants were required to respond to one out of two different questions: (1) which emotion she felt among 4 possible options (anger, shame, disgust and a neutral emotion) and (2) how much she focused on her physical appearance on a 4-point Likert scale (1 = not at all, 4 = a lot) (refer to Fig. 5 for trial structure and stimuli timing). The type of question was counterbalanced between the two blocks in such a way that participants responded to both the questions for each sentence at the end of the task. In each block, the objectifying and non-objectifying trials were balanced and randomly presented. Two versions of the experiment counterbalanced the association between the sets of faces and the objectifying and non-objectifying conditions. The faces within each set were randomly paired with the sentences in each condition, but always only uttered objectifying or non-objectifying sentences according to condition. Throughout the experiment, each face was presented eight times (4 per block), whereas each sentence was presented twice (once per block).

In each UG trial, each man’s face was presented and followed by the presentation of the associated offer, lasting on the screen until the participant's response. Subsequently, the outcome of the choice (i.e., how much the participant and the bidder received, respectively) was presented (see Fig. 5). There were five possible bidder-participant money partitions (5:5, 6:4, 7:3, 8:2, and 9:1 euros), repeated twice and randomly associated with the faces within each objectification condition (i.e., 10 trials per condition). Before the task, participants were informed that each offer was independent from each other and was preregistered during the fictitious experimental phase.

### EEG acquisition and pre-processing

The EEG signal was recorded with an eego sports system (ANT Neuro) from 64 Ag/AgCl shielded electrodes placed in the standard 10–10 locations on an elastic cap (Brain Products). The online reference was Cpz. The impedance in each electrode was kept below 20 KΩ, and the signal was amplified and digitized with a sampling rate of 1000 Hz. The pre-processing was performed with EEGlab^64^, a free toolbox of MATLAB. The raw signal of each participant was reduced to a sampling rate of 250 Hz, and a high-pass filter of 0.1 Hz (zero-phase FIR filter with cut-off frequency (− 6 dB): 0.05 Hz) was applied. The continuous data was re-referenced offline to the average of all electrodes, and segmented into face-centred epochs, ranging from 500 ms before to 1500 ms after face stimulus onset. Next, Independent Component Analysis (AMICA algorithm,^65^) was performed to remove both horizontal and vertical ocular artefacts, identified by IClabel tool^66^, an automatic EEG IC classification plugin of EEGlab. Further artefact rejection was conducted by removing noisy epochs in which the signal exceeded ± 70 µV in a smaller time range between − 200 ms and 800 ms using MATLAB toolbox ERPLAB^67^. Noisy epochs in this time-window were more than 15% in four participants, who were then excluded from the EEG analyses. Finally, a baseline of 200 ms pre-stimulus and a low-pass filter of 40 Hz were applied.

### Data analysis

All neural and behavioural analyses were performed using the IBM SPSS Statistics for Windows, version 25^68^.

For behavioural data of the OT, we computed the averages of the levels of self-objectification as a function of objectification condition (objectifying and non-objectifying contexts), and we compared them with a paired-sample t-test. In addition, we computed the response frequencies of each emotion as a function of each objectification condition, and we analysed them with a repeated measures ANOVA with emotion (anger, disgust, shame and neutral emotion) and objectification (objectifying and non-objectifying contexts) as within-participants factors. All pairwise comparisons were Bonferroni-corrected.

For the EEG data during the OT, ERP components, electrodes and time-windows of interest were determined based on previous studies^29–31,34,36,37,43,69^. For the N170, mean amplitude was selected in parietal electrodes (P7, P8) for a time range spanning from 175 to 225 ms. For the EPN, mean amplitude was computed over parieto-occipital electrodes (P7, P8, PO7, PO8, O1, O2) between 250 and 350 ms, whereas the LPP mean amplitude interested centro-parietal electrodes (Cpz, Cp1, Cp2, Cp3, Cp4), ranging from 500 to 800 ms. The effect of the context manipulation on the N170 was tested by a repeated measure ANOVA with objectification (objectifying, non-objectifying context) and lateralization (left, right) as within-participants factors (see^29,34,37,40^ for a right-lateralized N170). Two paired-sample t-tests, instead, were used to compare the objectifying vs. the non-objectifying context for the EPN and the LPP components.

In the UG task, the frequency of rejected offers (collapsed across the 5 different offers) was compared between the objectifying vs. non-objectifying context with a paired-sample t-test.

Finally, a first correlation analysis was performed between the level of self-objectification, the frequencies of negative emotion responses, and the frequencies of rejection, all in the objectifying condition (N = 36). Three further correlation analyses were performed between each ERP component (N170, EPN and LPP) and the behavioural measures in the OT (i.e., level of self-objectification and the frequency of negative emotions), still in the objectifying condition (N = 32). The multiple comparisons in each correlational analysis were controlled by applying false discovery rate (FDR) corrections to relevant *p*-values. All the reported significant correlations survived FDR corrections, otherwise an uncorrected *p*-value (*p*_uncorr_) is reported.

## Supplementary Information

Supplementary Information.

## Data Availability

All raw data can be access online (https://osf.io/vkqyr/).
