# Supplementary material for: Electrophysiological, emotional and behavioural responses of female targets of sexual objectification
Source: Sci Rep. 2023 Apr 8;13:5777. doi: 10.1038/s41598-023-32379-w (PMC10082788; doi:10.1038/s41598-023-32379-w)
Supplement: Supplementary file 1 — Supplementary Information. [file 41598_2023_32379_MOESM1_ESM.docx]

**Supplementary online materials S1**

**Who says What task:**

*Procedure*

The WSW task was administered to assess whether participants associated the male faces correctly to one of the two objectification conditions above chance level at the end of the OT. In each trial (20 in total), the face of each man was presented and followed by two sentences: An objectifying and a non-objectifying sentence only one of which the man had actually pronounced. Participants were asked to select which of the two sentences had been pronounced by the man in the previous task. They were forced to guess, if they did not remember if any of the sentences were pronounced by that specific male target.

*Data analysis*

For the WSW task, the percentage of accurate responses was calculated and averaged across participants. We also controlled whether a facilitation in the association for one of the two contexts occurred performing a paired-sample t-test between the percentage of accuracy in the objectifying vs the non-objectifying conditions.

*Results*

The mean percentage of the accuracy of the whole sample was 70%, assuring that the OT allowed participants to associate faces to a specific condition above chance level (50%). The percentage of accuracy between the objectifying vs non-objectifying condition did not differ, *t*(35) = 1.59, *p* = 0.12. The final correlational analyses reported in the main text were conducted including only those faces that were correctly recognized by participants (see below Supplementary online material, S2).

**Supplementary online material S2**

**The effect of punishment behaviours towards explicitly recognized objectifying men.**

In order to better assess whether the punishment behaviours in the UG were implicitly or explicitly endorsed by participants, we performed a supplementary repeated measures ANOVA on the frequency of rejections in the UG as a function of the Objectification (objectifying and non-objectifying condition) and the WSW accuracy (i.e., whether the faces in the WSW were correctly or incorrectly associated with the objectification condition).

Results showed that the main effect of Objectification, *F*(1,35) = 11.11, *p* = .002, *η_p_^2^* = .24, and the main effect of WSW accuracy, *F*(1,35) = 41.05, *p* < .001, *η_p_^2^* = .54, were qualified by a significant interaction, *F*(1,35) = 5.58, *p* = .024, *η_p_^2^* = .14. Pairwise comparisons showed that the frequency of rejections for objectifying men (*M* = 4.97, *SD* = 2.01) were higher than those for non-objectifying men (*M* = 3.81, *SD* = 2.07), p = .006, but only when the men had been correctly associated with the objectification condition. Indeed, no difference occurred in the frequency of rejections when the men had been incorrectly associated (obj_incorrected_: *M* = 1.83, *SD* = 1.32; non-obj_incorrected_: *M* = 2.11, *SD* = 1.30), *p* = .29 (see Figure S1).


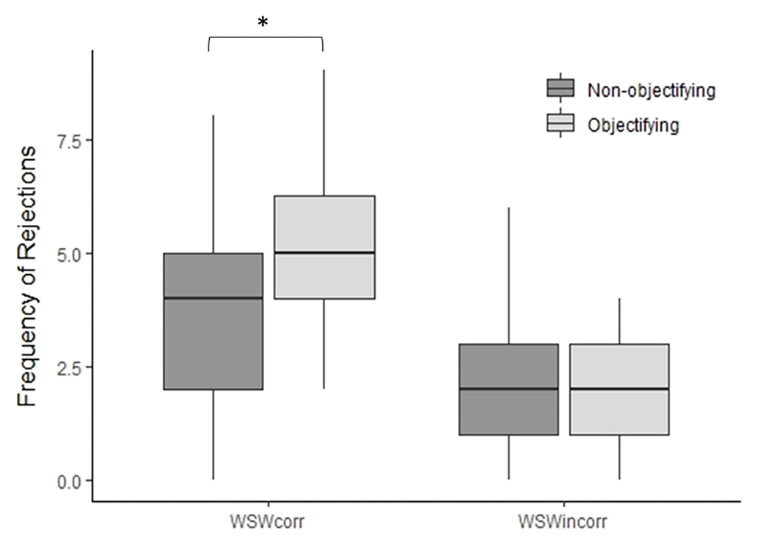


**Figure S1. Behavioural results of the Ultimatum Game for correctly and incorrectly remembered faces in the WSW**. Frequency of rejections as a function of WSW accuracy (faces correctly, WSWcorr, or incorrectly, WSWincorr, reported) and objectification condition (objectifying and non-objectifying context). Error bars = Standard error. (*) indicates significant differences (*p* < .05).

These results confirmed that the increased punishment behaviours specifically towards the objectifying men were explicitly endorsed by participants.

**Supplementary online material S3**

**Check for the objectification manipulation in the correlational results.**

In order to verify whether the patterns that emerged in the correlational analyses were specific to the objectification and did not occur in the non-objectification condition, we performed the same, complementary correlational analyses with the variables in the non-objectification condition. Again, multiple comparisons were controlled applying the FDR correction to all relevant p-values.

The results between the level of self-objectification, the extent of reporting negative emotions, and the frequency of rejection in the UG (*N*=36), only showed a significant positive correlation between the level of self-objectification and the frequency of anger, *r*(35) = .56, *p* < .001, and disgust *r*(35) = .62, *p* < .001.The results for each ERP and the behavioural measures in the OT showed no significant correlations (all *r* values ranged between -.19 to .19, *ps* > .05).

**Supplementary online materials S4**

**Pre-validation of verbal stimuli:**

At the beginning, 128 objectifying and 128 non-objectifying sentences were created. Objectifying sentences were inspired by “pick-up lines” (e.g., “Bambola, che gambe!”, direct English translation: “Baby, what legs!”) or typical “sexist stereotypes” (e.g., “Più la gonna è corta, meno la donna è intelligente”, direct English translation: “The shorter the skirt, the less intelligent the woman is”) and, in both cases, the focus was on woman’s aesthetic qualities. Non-objectifying sentences were created based on objectifying ones, and matched them in syntactic and grammatical terms. Importantly, in non-objectifying sentences there was no reference to aesthetic or physical aspects and sentences tended to be as neutral as possible (e.g., “Signora, che sonno!” direct English translation: “Miss, I am so sleepy!”, or “Più la storia è corta, meno i bambini sono interessati”, direct English translation: “The shorter the story, the less interested the children are”). In the pre-validation phase, 132 women (age: *M* = 29.6, *SD* = 1.3) were recruited by advertisement posted on social network sites to respond to an online questionnaire in which they were required to read the sentences and to answer to two questions that were presented in a random order: (i) whether they felt objectified (yes or not) and (ii) which emotion they experienced among 16 response options, randomly listed (anger, sadness, disgust, happiness, surprise, contempt, embarrassment, shame, satisfaction, gladness, proudness, calmness, neutrality, disinterest, estrangement). Objectifying sentences that reported 70% or more “yes” responses to the objectification question were retained in the original set (the corresponding non-objectifying phrases received 23% or less “yes” responses). Among the objectifying and non-objectifying sentences, the four most frequent reported emotions (between 12% and 34%) were anger, disgust, shame and neutrality (all the other emotions were reported less than 7%). The original set consisted in 104 sentences, half of which were objectifying and the other half were non-objectifying.

In the present study, we selected 84 sentences (42 objectifying and 42 non-objectifying) from the original set. Four of them (2 objectifying and 2 non-objectifying) served as stimuli for the practice trials, while the resulting 80 sentences (40 objectifying and 40 non-objectifying) served as stimuli for the experimental trials. The means of the length and frequency of the key-words in the objectifying (e.g., “legs” and “skirt”) vs. non-objectifying (“sleep” and “story”) sentences were compared on the basis of the “SUBTLEX-IT” dataset (available at the <http://crr.ugent.be/programs-data/subtitle-frequencies>), and they were not significantly different (in length: *t*(82) = .05, *p* = .96; and frequency: *t*(82) = .06, *p* = .95). The mean number of the words across the selected sentences was 8,36 (*SD* = 2,47) and did not differ between objectifying and non-objectifying sentences (*t*(82) = -.09, *p* = .93).

**Supplementary online materials S5**

**Cover story and task instructions provided to participants.**

During the recruitment, participants were told that the study aimed at investigating the neural correlates of emotional responses underlying specific social interactions between men and women. In the consent form, the specific social interactions referred to the sexual objectification manipulation, but no other information about the aims were reported, except for a simple description of each task.

The instructions of the first task (OT) asked participants to immerse in different social interactions imaging themselves to interact with several men for real. Participants were told that these men took part in a previous experimental phase of the experiment, during which their responses were registered (NB: the fictitious belief to interact with existent participants also showed to be in line with the typical instructions of the Ultimatum Game). Participants received no further information about the identities of the other supposed participants. The participants were informed that the social interactions consisted in the presentation of sentences that were directed to them, or generally to women (such as those of sexist stereotypes) - but always in their presence. These sentences had been pronounced by the men, the face of which was presented immediately after the sentence.

For the Ultimatum Game task, participants were told that each of the men met in the previous tasks had been asked to divide an amount of 10 euros between him and her. Participants’ task was to evaluate the division of money and to decide whether to accept or reject it. When participants would have accepted the money division, each of the players would have receive the amount expected. In the case of rejection, nobody would get anything. Participants were then informed that their final payment would have depended on the average of their three, randomly selected choices.
